# The development, reliability, and validity of the Facilitator Assessment Tool: An implementation fidelity measure used in Parenting for Lifelong Health for Young Children

**DOI:** 10.1111/cch.13075

**Published:** 2022-11-11

**Authors:** Mackenzie Martin, Jamie M. Lachman, Hugh Murphy, Frances Gardner, Heather Foran

**Affiliations:** ^1^ Department of Social Policy and Intervention University of Oxford Oxford UK; ^2^ MRC/CSO Social and Public Health Science Unit University of Glasgow Glasgow UK; ^3^ Institute for Psychology Alpen‐Adria‐University Klagenfurt Klagenfurt Austria

**Keywords:** behaviour, fidelity, implementation, parenting

## Abstract

**Background:**

The Parenting for Lifelong Health for Young Children (PLH‐YC) programme aims to reduce violence against children and child behaviour problems among families in low‐ and middle‐income countries (LMICs). Although the programme has been tested in four randomised controlled trials and delivered in over 25 countries, there are gaps in understanding regarding the programme's implementation fidelity and, more generally, concerning the implementation fidelity of parenting programmes in LMICs.

**Aims:**

This study aims to address these gaps by examining the psychometric properties of the PLH‐YC‐Facilitator Assessment Tool (FAT)—an observational tool used to measure the competent adherence of PLH‐YC facilitators. Examining the psychometric properties of the FAT is important in order to determine whether there is an association between facilitator competent adherence and programme outcomes and, if correlated, to improve facilitator performance. It is also important to develop the implementation literature among parenting interventions in LMICs.

**Methods:**

The study examined the content validity, intra‐rater reliability, and inter‐rater reliability of the FAT. Revision of the tool was based on consultation with programme trainers, experts, and assessors. A training curriculum and assessment manual was created. Assessors were trained in Southeastern Europe and their assessments of facilitator delivery were analysed as part of a large‐scale factorial experiment (*N* = 79 facilitators).

**Results:**

The content validity process with PLH‐YC trainers, experts, and assessors resulted in substantial improvements to the tool. Analyses of percentage agreements and intraclass correlations found that, even with practical challenges, assessments were completed with adequate yet not strong intra‐ and inter‐rater reliability.

**Conclusions:**

This study contributes to the literature on the implementation of parenting programmes in LMICs. The study found that the FAT appears to capture its intended constructs and can be used with an acceptable degree of consistency. Further research on the tool's reliability and validity—specifically, its internal consistency, construct validity, and predictive validity—is recommended.

**Key Messages**

- The Facilitator Assessment Tool (FAT) is an observational implementation measure used to assess the competent adherence with which programme facilitators deliver the Parenting for Lifelong Health‐Young Children (PLH‐YC) programme.
- As PLH‐YC is being delivered widely in many countries, it is necessary to establish the tool's psychometric properties to provide confidence that the competent adherence of facilitators is consistently and accurately assessed.
- This study analysed the content validity, intra‐rater reliability, and inter‐rater reliability to provide initial evidence of the tool's ability to capture its intended constructs and be used consistently by and between assessors.
- Further analyses of the FAT's reliability and validity (internal consistency, construct validity, and predictive validity) are warranted and would provide additional evidence as to the strength of this tool for measuring facilitator competent adherence.

## 1 INTRODUCTION

### 1.1 Parenting for Lifelong Health

There is considerable evidence that parenting programmes increase positive parenting and parent–child relationships as well as reduce child maltreatment and behaviour problems (e.g., Chen & Chan, 2016; Furlong et al., 2013). One such programme for families with children across the developmental spectrum is the Parenting for Lifelong Health for Young Children programme (PLH‐YC), which was originally developed in South Africa by individuals from the Universities of Bangor, Cape Town, and Oxford in collaboration with the World Health Organization, UNICEF, and Clowns Without Borders South Africa along with input from parents and practitioners (Lachman, Sherr, et al., 2016). PLH‐YC is a group‐based parenting programme for parents of children ages 2 to 9 years rooted in social learning theory and behaviour change principles (e.g., goal setting and discussing progress on goals) (Lachman, Sherr, et al., 2016; Michie et al., 2015; Ward et al., 2014). The programme ranges from five to 12 sessions in length and uses participatory, nondidactic, and strengths‐based approaches to empower parents to develop skills in fostering positive relationships, handling conflict and emotions, and employing effective disciplinary approaches (Lachman, Sherr, et al., 2016). Parenting for Lifelong Health (PLH) programmes are now being implemented in over 25 countries in Africa, Asia, and Southeastern Europe. To date, four randomised controlled trials (RCTs) have examined the effectiveness of PLH‐YC—two in South Africa, one in the Philippines, and one in Thailand (Gardner et al., forthcoming; Lachman et al., 2017, 2021; Ward et al., 2020). These studies found improved positive parenting and child behaviour, while reducing family violence. Further information about PLH and its growing evidence base is available in numerous published papers, protocols, and resources (e.g., Martin, Lachman, et al., 2021; Shenderovich et al., 2020; World Health Organisation [WHO], 2020). As the evidence supporting parenting programmes such as PLH is positive and substantial, studying their implementation fidelity represents an important way to explore which programme elements are correlated with outcomes and how to improve delivery.

### 1.2 Measures of competent adherence

Many parenting programmes have developed programme‐specific tools to measure implementation fidelity (e.g., Martin, Steele, et al., 2021). This paper focuses on a measure developed for and used in PLH‐YC to assess two aspects of implementation fidelity—competence and adherence, which together capture the skill and diligence with which facilitators deliver intervention components (Fixsen et al., 2005). Assessing competent adherence has numerous research and practical benefits, including providing an objective assessment of the extent to which a particular parenting programme is implemented as planned. These assessments allow researchers to examine whether facilitator delivery is associated with parent and/or child outcomes, understand what makes an effective facilitator, and indicate where to target programme improvements (Forgatch et al., 2005).

A systematic review by Martin, Steele, et al. (2021) identified 65 measures of competent adherence employed in 63 parenting programmes. Parenting programmes differ according to content and delivery, and this variety is reflected in the number of fidelity tools available. Measures also vary in other respects: Some use observational, nonobservational, or a combination of methods; some are structured with dichotomous or Likert scale items; and all capture one or more aspects of competent adherence.

### 1.3 PLH facilitator training and assessment

Facilitators—typically community members and professionals including teachers, psychologists, and social workers—receive PLH‐YC training through a 5‐day workshop (approximately 30 h). Training is provided by Clowns Without Borders South Africa (CWBSA), a nonprofit serving as a capacity building agency for the dissemination of PLH. In training, facilitators learn to implement programme activities and skills, including using group discussions on parenting challenges, illustrated comics to identify and model parenting skills, and group activities to practice skills (Lachman, Cluver, et al., 2016). Following training, facilitators deliver PLH‐YC and receive regular supervision from coaches. The PLH‐YC Facilitator Assessment Tool (FAT) was developed by study investigators and programme developers to assess the quality with which facilitators deliver programme‐specific activities and techniques.

### 1.4 PLH facilitator assessment procedure

The FAT assessment procedure is similar to other measures of facilitator quality of delivery, including measures used by Parent Management Training‐Oregon Model (PMTO) (Holtrop et al., 2020; Knutson et al., 2019) and Incredible Years (IY) (Eames et al., 2008). Like the PMTO and IY tools, the FAT uses observational methods (live or video‐recorded) to assess facilitator delivery of entire programme sessions. Observational methods are used since these are considered more objective than nonobservational methods such as facilitator self‐reports, which could be prone to social desirability bias (Eames et al., 2008; Stone et al., 1999). However, observational assessments are often more resource‐intensive and may be susceptible to reactivity bias (Girard & Cohn, 2016). Training of FAT assessors is approximately 14 h and includes theoretical and practical components following a training curriculum, assessor manual, and coding matrix. As the training is conducted in low‐income settings, fewer financial resources are dedicated to training assessors than in high‐income settings.

### 1.5 FAT

The FAT is composed of two subscales (50 items). The Activities Subscale assesses facilitator adherence to three key PLH‐YC activities (24 items)—home activity discussion (10 items; e.g., “identify specific challenges when shared by at least one parent”), illustrated story discussion (seven items; e.g., “discuss possible solutions for negative stories”), and group practice activity (seven items; e.g., “debrief with the participants about experiences and feelings”). The Skills Subscale assesses facilitator competence in delivering key PLH‐YC process skills (26 items)—modelling parenting behaviours (seven items, e.g., “give positive, specific, and realistic instructions”), demonstrating collaborative facilitation (seven items; e.g., “accept participant responses verbally by reflecting back what the participant says”), encouraging participation (seven items; e.g., “participants appear comfortable and involved in the session”), and utilising leadership skills (six items, e.g., “use open‐ended questions during group discussions”). Items are rated using a 4‐point Likert scale (0 = *inadequate*, 1 = *needs improvement*, 2 = *good*, 3 = *excellent*). Items are summed to produce an impression score represented as a percentage.

### 1.6 Current study

Although there is growing evidence of the effectiveness of PLH‐YC, there are gaps in understanding regarding its implementation quality and, more specifically, concerning facilitator competent adherence. As there was not a suitable measure of competent adherence available in the literature, this study describes the development of the tool used to assess PLH‐YC facilitators and provides preliminary psychometric evidence on the tool using data from the delivery of PLH‐YC in North Macedonia, the Republic of Moldova (“Moldova”), and Romania as part of the RISE study. RISE is a collaboration seeking to evaluate the effectiveness and costs of PLH‐YC (Frantz et al., 2019; Lachman et al., 2019). As a result, this study aimed to answer the following research question: what is the content validity, intra‐rater reliability, and inter‐rater reliability of the FAT as a measure of PLH‐YC facilitator competent adherence in Southeastern Europe? As the first study on the FAT's psychometric properties, these indices were selected to examine whether the tool shows promise for further psychometric analyses or needs revision.

## 2 METHODS

This paper examined the content validity, intra‐rater reliability, and inter‐rater reliability of the FAT in Southeastern Europe using COSMIN recommendations (Mokkink et al., 2010). Content validity was examined by consulting with three stakeholder groups and revising the FAT accordingly. Intra‐ and inter‐rater reliability was examined by determining the degree to which assessors used the FAT consistently over time as well as with each other.

### 2.1 Participants

This study involved three stakeholder groups. First, during the content validity process, we consulted with eight certified trainers from CWBSA with at least 2 years of experience conducting FAT assessments in numerous countries. Second, we sought the advice of three parenting programme experts. These experts were consulted due to their extensive knowledge of PLH‐YC and on conducting facilitator assessments in both research and practice. Third, those who conducted facilitator assessments (“assessors”) supported our evaluation of all three psychometric properties. The assessors were 11 trained coaches involved in RISE (*n* = 5 in Moldova*, n* = 3 in North Macedonia, and *n* = 3 in Romania). The Moldovan assessors had a range of experiences and backgrounds supporting vulnerable families, including teaching and family therapy. The North Macedonian assessors were later career psychologists with experience in conducting assessments similar to the FAT. The Romanian assessors were early career psychologists based at a local university. The Moldovan and Romanian assessors also had prior experience as PLH‐YC facilitators.

### 2.2 Procedure and analytic strategy

#### 2.2.1 Content validity

The FAT's content validity—the extent the tool appears to capture the intended constructs—was examined by gathering and synthesising the perspectives of the three aforementioned stakeholder groups on the measure's comprehensiveness, relevance, and comprehensibility (Mokkink et al., 2010; Terwee et al., 2018). First, we held a content validity workshop with CWBSA trainers, wherein detailed field notes were taken. The trainers were asked to describe their use of the FAT; share their perspectives on the tool's comprehensiveness, comprehensibility, and relevance; and suggest revisions to improve the tool's utility and accuracy. Their feedback was used to modify the FAT and create an initial training curriculum and manual. Second, we consulted the three parenting programme experts, who provided feedback on an updated version of the FAT and training manual. Third, during assessor training, RISE study assessors provided input on the FAT's comprehensiveness and relevance. It is important to note that the assessor training was not delivered to completion in North Macedonia and Romania due to scheduling conflicts, which may have compromised assessment reliability.

#### 2.2.2 Intra‐rater reliability

Intra‐rater reliability, or assessor consistency, was examined by having each assessor observe a video recording of a facilitator delivering the programme twice with assessments conducted 3 weeks apart (Gwet, 2014; Heinl et al., 2016). As a result, a video from one facilitator per country was selected. It was only possible to assess one facilitator per country due to time and resource constraints. FAT scores were compared by calculating percentage agreements and intra‐class correlations (ICCs) for each assessor and subscale (Margolin et al., 1998). Percentage agreements were selected because they indicate the ratio of instances wherein assessors chose the same rating. Agreement levels above 70% were acceptable (Aspland & Gardner, 2003). ICCs were also examined to take chance agreement and correlation into account (Bruton et al., 2000; Koo & Li, 2016). A two‐way mixed‐effects model with an absolute agreement definition and single‐rater type was used (McGraw & Wong, 1996; Shrout & Fleiss, 1979). ICCs were interpreted within a 95% confidence interval where ICCs under 0.50 were considered poor, between 0.50 and 0.75 were moderate, between 0.75 and 0.90 were good, and above 0.90 were excellent (Koo & Li, 2016). Percentage agreements and ICCs were calculated using the “irr package” in R (Gamer et al., 2017). As all items of the FAT should have been completed, mean imputation was used to take missing data into account when less than 10% of the data was missing (Watkins, 2018).

#### 2.2.3 Inter‐rater reliability

Inter‐rater reliability, the degree to which different coders similarly assess facilitator delivery (Chen & Krauss, 2004; Cho, 1981), was examined by having assessors observe video recordings of the same three facilitators selected out of 31 possible facilitators in Moldova, 16 in North Macedonia, and 32 in Romania (Hallgren, 2012). Thus, videos from a total of nine facilitators were used. It was only possible to assess three facilitators per country due to time and resource constraints. The data were analysed using the same methods as the assessments of intra‐rater reliability.

## 3 RESULTS

### 3.1 Content validity

The content validity consultations produced recommendations to improve the FAT. CWBSA trainers made four recommendations: break up complex items into separate, simple items; use specific definitions for each item and Likert point; add items to capture missing activities and skills; and remove redundant items.

After revisions based on trainer feedback, the FAT was shared with the parenting programme experts. Rooted in evidence linking praise and reflexive statements to participant outcomes (Eames et al., 2010), the experts recommended adding three items to measure the frequency of specific and unspecific praise (i.e., expressing approval and appreciation) and reflexive statements (i.e., reiterating participant contributions).

The further revised tool was then shared with PLH‐YC coaches during assessment training. Initially, assessors recommended changes to item wording and suggested examples to include in the definitions. After using the tool, the assessors provided further insight, indicating which items were difficult to understand and how they could be improved. This process helped ensure clear differences between points on the Likert scale.

Following assessor input, the revised FAT was finalised, resulting in 62 items with 26 items in the Activities Subscale, 33 items in the Skills Subscale, and three items in the Frequency Subscale. A summary of the recommendations and changes made is provided in Table 1.

**TABLE 1:** Summary of recommendations and changes to FAT

Recommendation to improve the FAT	Stakeholder group	Changes made or example of changes made
Break up complex items into separate, simple items	CWBSA Trainers	They recommended that the item, “Did the facilitator accept participant responses verbally and physically?” be divided into four items to capture whether the facilitator demonstrated physical acceptance (e.g., nodding), verbal acceptance (e.g., “mhm”), openness (e.g., “Interesting suggestion!”), and use of a reflexive statement (e.g., “Am I understanding you to say that you will schedule daily time to play with your child?”).
Use specific definitions for each item and Likert point	CWBSA Trainers	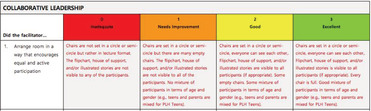
Add items to capture missing activities and skills	CWBSA Trainers	“Did the facilitator identify core building blocks connected to the story?” was added to the illustrated story items in the Activities Subscale.
Remove redundant items	CWBSA Trainers	The trainers recommended deleting the item, “Did the facilitator provide frequent praise throughout the discussion?” since praise was already incorporated into many questions.
Create the frequency subscale	Parenting Programme Experts	Three additional items were added to the FAT: “Please record the number of discrete times the facilitator used reflexive statements and praise (specific/unspecific) during first twenty minutes of the home activity discussion: (1) reflexive statements, (2) specific praise, and (3) unspecific praise.”
Changes to item wording	RISE Assessors	The item “Accepts parent responses physically” was changed to “Uses body language to show acceptance.”
Proposed examples to include in item definitions	RISE Assessors	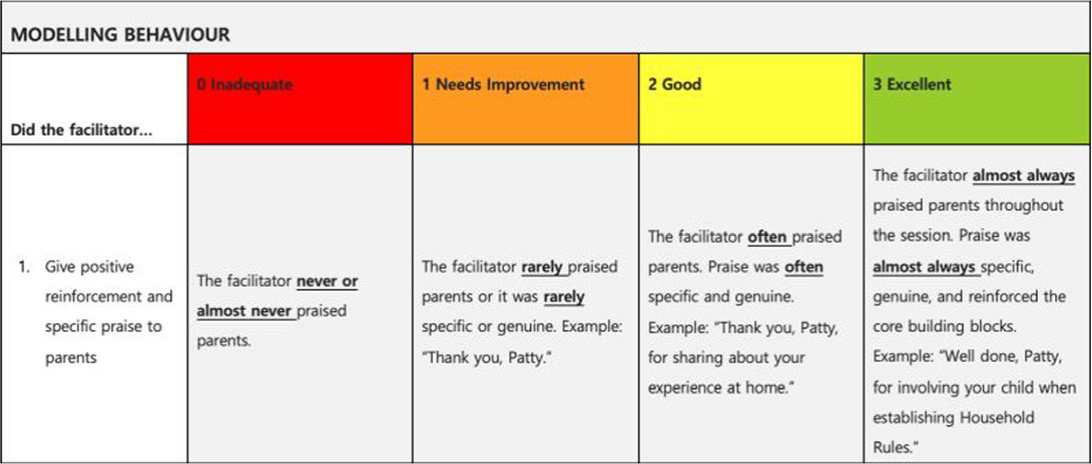

### 3.2 Intra‐ and inter‐rater reliability

In terms of intra‐rater reliability, the overall percentage agreements across the three countries ranged from 57.6% to 91.5% with ICCs of 0.52–0.94 (Tables 2 and 3). Subscale percentage agreements ranged from 46.2% to 92.3% with ICCs of 0.32–0.96 on the Activities Subscale, 45.5% to 90.9% with ICCs of 0.13–0.93 on the Skills Subscale, and 0.0% to 100.0% on the Frequency Subscale with ICCs of −0.79–1.00. At the country‐level, overall percentage agreements ranged from 57.6% to 89.9% with ICCs of 0.52–0.94 in Moldova, 78.0% to 88.1% with ICCs of 0.82–0.90 in North Macedonia, and 66.1% to 91.5% with ICCs of 0.79–0.94 in Romania. There was no missing intra‐rater reliability data.

**TABLE 2:** Assessor‐level intra‐rater reliability percentage agreements

Assessor and country	Total percentage agreement (activities and skills subscale, *N* = 59 items)	Activities subscale (*N* = 26 items) percentage agreement	Skills subscale (*N* = 33 items) percentage agreement	Frequency subscale (*N* = 3 items) percentage agreement
Moldova	57.6–89.9% (mean: 75.3%)	46.2–92.3% (mean: 74.6%)	45.5–90.9% (mean: 75.8%)	0.0–100.0% (mean: 60%)
North Macedonia	78.0–88.1% (mean: 83.1%)	80.8–92.3% (mean: 87.2%)	75.8–84.8% (mean: 79.8%)	0.0–100.0% (mean: 44.4%)
Romania	66.1–91.5% (mean: 76.8%)	69.2–92.3% (mean: 80.8%)	63.6–90.9% (mean: 73.7%)	66.7–100.0% (mean: 88.9%)

*Note*: Timepoint 1: December 2019, Timepoint 2: January 2020.

**TABLE 3:** Assessor‐level intra‐rater reliability ICCs

Country	Total score	Activity	Skills	Frequency
Moldova	0.52–0.94 (mean: 0.78)	0.32–0.94 (mean: 0.79)	0.13–0.93 (mean: 0.70)	−0.13‐1.00 (mean: 0.75)
North Macedonia	0.82–0.90 (mean: 0.86)	0.90–0.96 (mean: 0.93)	0.58–0.68 (mean: 0.62)	−0.79‐1.00 (mean: 0.30)
Romania	0.79–0.94 (mean: 0.85)	0.81–0.95 (mean: 0.86)	0.79–0.93 (mean: 0.84)	0.80–1.00 (mean: 0.93)

*Note*: Model: two‐way mixed effects, type: single rater, definition: absolute agreement.

In terms of inter‐rater reliability, the overall percentage agreements ranged from 18.1% to 74.0% with ICCs of 0.49–0.91 (Table 4, 5, 6). The overall percentage agreement across the three assessments was 18.1% (activities: 29.5%, skills: 9.1%, frequency: 0.0%) in Moldova with an ICC of 0.62 (activities: 0.71, skills: 0.37, frequency: 0.52), 74% (activities: 71.8%, skills: 75.8%, frequency: 11.1%) in North Macedonia with an ICC of 0.91 (activities: 0.93, skills: 0.86, and frequency: 0.95), and 32.8% (activities: 38.7%, skills: 33.3%, frequency: 22.2%) in Romania with an ICC of 0.49 (activities: 0.65, skills: 0.50, frequency: 0.84). There was no missing inter‐rater reliability data in Macedonia and a low amount of missing data in Moldova (0.11%) and Romania (4.30%). Assessors indicated that some data were missing as it was not possible to assess some items (e.g., an opportunity did not arise for the facilitator to deliver a programme component). These items should have been scored as each FAT item captures an aspect of PLH‐YC that should be delivered.

**TABLE 4:** Moldova inter‐rater reliability data by assessment

Facilitator assessment	Total score		Activity		Skills		Frequency	
	Percentage agreement	ICCs (95% CI)	Percentage agreement	ICCs (95% CI)	Percentage agreement	ICCs (95% CI)	Percentage agreements	ICCs (95% CI)
Moldova	18.1%	0.62 95% CI [0.53, 0.70]	29.5%	0.71 95% CI [0.62, 0.79]	9.1%	0.37 95% CI [0.25–0.49]	0.0%	0.52 95% CI [0.15, 0.89]
1	30.5%	0.77 95% CI [0.68–0.84]	50.0%	0.85 95% CI [0.76, 0.92]	15.2%	0.32 95% CI [0.17, 0.51]	*MISSING*	*MISSING*
2	15.3%	0.56 95% CI [0.40, 0.70]	23.1%	0.62 95% CI [0.43, 0.78]	9.1%	0.35 95% CI [0.17, 0.54]	0.0%	0.45 95% CI [−0.09, 0.98]
3	8.5%	0.49 95% CI [0.34, 0.64]	15.4%	0.57 95% CI [0.38, 0.75]	3.0%	0.35 95% CI [0.18, 0.54]	0.0%	0.63 95% CI [0.16, 0.99]

*Note*: ICC = intra‐class correlation, 95% CI = 95% confidence interval; model: two‐way mixed effects, type: single rater, definition: absolute agreement, five assessors and three facilitator assessments were used, missing: frequency data for assessment 1.

**TABLE 5:** North Macedonia inter‐rater reliability data by assessment

Facilitator assessment	Total score		Activity		Skills		Frequency	
	Percentage agreement	ICCs (95% CI)	Percentage agreement	ICCs (95% CI)	Percentage agreement	ICCs (95% CI)	Percentage agreements	ICCs (95% CI)
Overall	74.0%	0.91 [0.88, 0.93]	71.8%	0.93 [0.90, 0.95]	75.8%	0.86 [0.82–0.90]	11.1%	0.95 [0.85, 0.99]
1	71.2%	0.90 [0.84, 0.93]	69.2%	0.93 [0.86–0.97]	72.7%	0.84 [0.74–0.91]	33.3%	0.80 [0.21, 0.99]
2	71.2%	0.90 [0.84, 0.93]	69.2%	0.92 [0.86, 0.96]	72.7%	0.84 [0.74, 0.91]	0.0%	0.99 [0.90, 1.00]
3	79.7%	0.93 [0.89, 0.95]	76.9%	0.94 [0.90, 0.97]	81.8%	0.85 [0.75, 0.92]	0.0%	0.78 [0.20, 0.99]

*Note*: ICC = intra‐class correlation, 95% CI = 95% confidence interval; model: two‐way mixed effects, type: single rater, definition: absolute agreement; three assessors and three facilitator assessments were used.

**TABLE 6:** Romania inter‐rater reliability data

Facilitator assessment	Total score		Activity		Skills		Frequency	
	Percentage agreement	ICCs (95% CI)	Percentage agreement	ICCs (95% CI)	Percentage agreement	ICCs (95% CI)	Percentage agreements	ICCs (95% CI)
Overall	32.8%	0.49 [0.39, 0.59]	39.7%	0.65 [0.53, 0.75]	33.3%	0.50 [0.36, 0.63]	22.2%	0.84 [0.59, 0.96]
1	42.4%	0.64 [0.51, 0.75]	46.2%	0.72 [0.54, 0.85]	39.4%	0.57 [0.38, 0.74]	0.0%	0.71 [0.06, 0.99]
2	25.4%	0.47 [0.28, 0.63]	30.8%	0.55 [0.29, 0.75]	21.2%	0.40 [0.18, 0.62]	33.3%	0.90 [0.50, 1.00]
3	40.7%	0.62 [0.39, 0.77]	42.3%	0.71 [0.41, 0.86]	39.4%	0.54 [0.30, 0.73]	33.3%	0.92 [0.51, 1.00]

*Note*: ICC = intra‐class correlation, 95% CI = 95% confidence interval; model: two‐way mixed effects, type: single rater, definition: absolute agreement; three assessors and three facilitator assessments were used; missing: 24 items.

## 4 DISCUSSION

This study analysed three psychometric properties of the FAT. The content validity process resulted in a revised FAT that was more understandable, specific, and practical due to stakeholder recommendations to the FAT's items and assessment procedure. The analysis of assessor intra‐rater reliability found somewhat acceptable levels of consistency (overall percentage agreements ranged from 57.6% to 91.5% with ICCs of 0.52–0.94 and assessor inter‐rater reliability ranged from 18.1% to 74.0% with ICCs of 0.49–0.91). Although assessors did not always achieve consensus, the ICCs were, with few exceptions, larger than the percentage agreements and most exceeded the suggested cut‐off of ICC > 0.50 for moderate levels of reliability (Stemler & Tsai, 2008). This finding suggests that assessors were largely consistent in their application of the measure and its items, yet it was still difficult for assessors to achieve consensus on many occasions. It was particularly difficult for assessors from all countries to achieve intra‐ and inter‐rater reliability on the Frequency Subscale. Negative ICCs were found in multiple instances, indicating more within variance than between. As a result, this subscale was not rated reliably and should be dropped or modified.

### 4.1 Contextual factors

The intra‐ and inter‐rater reliability results are encouraging in light of challenges encountered during assessor training and varying levels of assessor experience. The training was not delivered to completion in North Macedonia and Romania due to scheduling conflicts, resulting in approximately 80% of the training being delivered in each of these contexts. In North Macedonia and Romania, three and two assessors respectively missed several hours of training due to other organisational commitments scheduled at the same time. Aside from Romania, the training relied on translation into local languages, which may have led to an imprecise understanding of assessment procedures. Further, there may be different levels of reliability across countries due to differences in assessment experience. Results may have been stronger in North Macedonia as these assessors were experienced psychologists accustomed to completing assessments similar to the FAT. In contrast, the Moldovan assessors, where reliability was lower, were less experienced practitioners from non‐psychology backgrounds. Thus, future training should take prior experience into consideration and provide additional support for those with less experience. Furthermore, future research should explore what assessor training, characteristics, skills, and ongoing supports are required for reliably conducting facilitator assessments.

### 4.2 Limitations and strengths

In addition to challenges delivering the training, this study had limitations. First, the study used a small sample (Koo & Li, 2016), necessitating caution in interpreting study results. Second, due to the study's limited scope, we used a purposive selection of sessions for assessment instead of random selection. This may have resulted in the assessment of nonrepresentative facilitator delivery (Walton et al., 2017). Third, missing data may have compromised results. Future revisions of the FAT should ensure that assessors complete all items using strategies such as highlighting the importance of completing all items in assessor training and establishing monitoring and evaluation processes wherein FAT forms are checked for completion.

Despite limitations, this study makes an important contribution to our understanding of the psychometric properties of an assessment tool widely used to assess facilitators of an evidence‐based parenting programme in LMICs. While valuable in all contexts, the need for practical, reliable, and valid assessment tools in low‐income contexts is particularly heightened. The study found sufficient intra‐ and inter‐rater reliability despite challenges encountered during training. However, the findings suggest that more attention should be paid to how reliability can be strengthened and explore whether those with certain backgrounds and skills would be better suited to conducting reliable assessments. For instance, future assessor training could require assessors to achieve an acceptable minimum level of intra‐ and inter‐rater reliability prior to conducting FAT assessments in practice. Further, two common issues in studying observational measures were avoided. First, facilitator reactivity to assessment was minimised because all programme sessions were recorded, thereby decreasing the likelihood that facilitators performed differently for assessments (Kazdin, 1982). Second, assessors did not evaluate facilitators they supervised to ensure assessor independence from the results (Walton et al., 2017).

## 5 CONCLUSION

The results of this study suggest that the Facilitator Assessment Tool appears to capture the competent adherence with which facilitators deliver Parenting for Lifelong Health for Young Children. Future research is necessary to strengthen the reliability of the measurement due to results suggesting sufficient though not high levels of intra‐ and inter‐rater reliability. We also recommend further research on the FAT's psychometric properties. These include research on its internal consistency to determine whether Cronbach's alphas and omegas indicate that items are appropriately associated with each other, construct validity to explore whether the tool is measuring its intended constructs via exploratory factor analyses, and predictive validity to examine whether higher facilitator competent adherence scores are associated with improved family outcomes (Barchard, 2010; Markus & Lin, 2010; Mislevy & Rupp, 2010). Relatedly, given that over 3000 facilitators are delivering PLH programmes internationally across more than 25 countries, we recommend that the FAT's psychometric properties be examined across multiple contexts. We also recommend that similar analyses are conducted of the PLH programme for adolescents, which has similar delivery methods but involves different activities from PLH‐YC. In summary, this study was an important first step in ensuring reliable assessments of programme delivery—a critical factor when monitoring the dissemination and scale‐up of evidence‐based interventions.

**Abbreviations**
COSMIN: COnsensus‐based standards for the selection of health Measurement INstruments
CWBSA: Clowns Without Borders South Africa
FAT: Facilitator Assessment Tool
ICC: intraclass correlation
IY: incredible years
PLH: Parenting for Lifelong Health
PLH‐YC: Parenting for Lifelong Health‐Young Children
PMTO: Parent Management Training‐Oregon Model
RCTs: randomised controlled trials

## CONFLICT OF INTEREST

MM, HM, and HMF declare that they have no competing interests. JML and FG are co‐developers of PLH for Young Children (licensed under a Creative Commons 4.0 Non‐commercial No Derivatives license) and, with colleagues, co‐founders of the Parenting for Lifelong Health initiative. JML receives occasional fees for providing training and supervision to facilitators and coaches. JML and FG have participated (and are participating) in a number of research studies on the programme as investigators and the University of Oxford receives research funding for these studies. Conflict is avoided by declaring potential conflicts and by conducting and disseminating rigorous, transparent, and impartial evaluation research on both PLH and other similar parenting programmes.

## ETHICS STATEMENT

The research received approval from the University of Oxford's Department of Social Policy and Intervention Research Ethics Committee (Ref: SPIC1a_20_0004), the University of Klagenfurt Ethics Board of the Institute of Psychology (Ref: 2018‐21), as well as by local country institutional review boards in North Macedonia (Ref: 03‐1475‐2), the Republic of Moldova (Ref: 43‐56/12.04.018), and Romania (Ref: 322/1.03.2019).

## Data Availability

The Parenting for Lifelong Health for Young Children‐Facilitator Assessment Tool is included as supplementary material.

## Appendix A FACILITATOR ASSESSMENT TOOL

Parenting for Lifelong Health for Young Children ‐Facilitator Assessment Tool (PLH‐YC‐FAT)

**Table:**

Assessor name:			
Facilitator name:		Facilitator ID:	
Assessment date:		Session number and date:	
Video file name:		Session/video length:	
Number of enrolled parents:		Number of parents in attendance:	
Facilitator age:		Facilitator gender:	
Has the facilitator been assessed before (Y/N)?		If yes, how many times has the facilitator been assessed previously?	
Co‐facilitator name:		Facilitator condition	

ACTIVITY SUB‐SCALE

**Table:**

HOME ACTIVITY DISCUSSION	Inadequate 0	Needs improvement 1	Good 2	Excellent 3
SESSION NUMBER:________________________ START TIME ON VIDEO: _________________ END TIME ON VIDEO:_____________________				
Remind parents of the core home activity for the previous week at the beginning of the activity				
Review core building blocks from previous session with parents at the beginning of the activity				
Parents share experiences of how home activity went during the week				
Keep parents focused on core home activity				
Help parents connect experiences to the core building blocks				
Explore at least one specific challenge experienced by a parent regarding the main home activity				
Explore solutions to challenge shared				
Help parents choose an appropriate and specific solution				
At least one parent practice solutions to challenges **OR** ways to improve parenting skills				
Debrief with the parents after practicing solutions to challenges				
Praise and encourage parents to try solution at home				
Thank and praise parents for sharing experiences (at the end of the home activity discussion)				
Comments/notes:				

FREQUENCY SUB‐SCALE

Please count the number of discrete times the facilitator used reflexive statements and praise (specific/unspecific) during the FIRST 20 MINUTES OF THE HOME

**Table:**

Behaviour	Frequency	
**Reflexive Statements**		
**Praise**	**Unspecific:**	**Specific**

ILLUSTRATED STORY DISCUSSION	Inadequate 0	Needs Improvement 1	Good 2	Excellent 3
SESSION NUMBER:________________________ START TIME ON VIDEO: __________________ END TIME ON VIDEO: _____________________				
Read through story with parents				
Explore actions, behaviours, and emotions				
Make sure that the questions in the manual have been covered				
Identify core building blocks connected to the story				
Comments/notes:				

GROUP PRACTICE	Inadequate 0	Needs improvement 1	Good 2	Excellent 3
SESSION NUMBER:_______________ START TIME ON VIDEO:____________ END TIME ON VIDEO:________________				
**BIG GROUP PRACTICE**				
Establish roles for big group practice (e.g. parent and child)				
Set up scenario and use space appropriately				
Describe exactly what “parent” and “child” will be doing during the group practice				
Give support to parents during group practice (shadow)				
Debrief with “parent” about experiences and feelings				
Debrief with “child” about their experience and feelings				
Thank and praise parents who practiced in big group				
**SMALL GROUP PRACTICE**				
Practice in pairs while supporting around room				
Debrief with parents after practicing in pairs				
Thank and praise parents				
Comments/notes:				

SKILLS SUB‐SCALE

**Table:**

MODELLING BEHAVIOUR	Inadequate 0	Needs Improvement 1	Meets Expectations 2	Exceeds Expectations 3
Give lots of positive reinforcement and specific praise to parents				
Give positive, specific, and realistic instructions				
Maintain commitments to time management principles				
Model behaviours with co‐facilitator				
Demonstrate respectful behaviour towards parents				
Comments/notes:				

ACCEPT‐EXPLORE‐CONNECT‐PRACTICE	Inadequate 0	Needs Improvement 1	Meets Expectations 2	Exceeds Expectations 3
**ACCEPT**				
Uses body language to show acceptance				
Accepts parent responses verbally				
Openness				
Reflexive statements				
**EXPLORE**				
Explore experience/opinion of parent in detail using open‐ended questions				
Explore thoughts and feelings				
Explore child perspective				
**CONNECT**				
Connect experiences to the building blocks from session				
Connects individual experiences to universal principles				
**PRACTICE**				
Identify opportunities to practice skills (in addition to the structured group practice)				
Comments:				

COLLABORATIVE LEADERSHIP	Inadequate 0	Needs Improvement 1	Meets Expectations 2	Exceeds Expectations 3
Arrange room in a way that encourages equal and active participation				
Facilitator is situated within the group, is at the level of the parents, and in a different place than the co‐facilitator				
Parents appear engaged in session				
Parents appear comfortable and satisfied				
Parents share views and opinions				
Parent‐facilitator participation ratio				
Assure equal and active participation among parents				
Targeted engagement of quiet or non‐participating parents				
Limiting parent responses				
Keep parents on topic of discussion				
Demonstrate knowledge of session content				
Level of self‐confidence with session content				
Help parents generate their own ideas regarding principles or solutions to challenges				
Help parents assess consequences of proposed solutions				
Allow parents to choose their own solutions to challenges				
Make sure that solutions are positive, specific, and realistic				
Maintain leadership and control of the group				
Work well with the co‐facilitator				
Comments:				

OVERALL ASSESSMENT

**Table:**

Activities assessment		Skills assessment	
Total score on core activities (A)		Total score core facilitation skills (C)	
Total possible score (B)	**78**	Total possible score (D)	**99**
Total percent score core activities = (A/B) × 100%	**%**	Total percent score core skills = (C/D) × 100%	**%**
**What are the facilitator's strengths?**			
**What does the facilitator need to improve?**			
**Recommendations:**			
